# Spread and Transmission of Bacterial Pathogens in Experimental Populations of the Nematode Caenorhabditis elegans

**DOI:** 10.1128/AEM.01037-14

**Published:** 2014-09

**Authors:** S. Anaid Diaz, Olivier Restif

**Affiliations:** Disease Dynamics Unit, Department of Veterinary Medicine, University of Cambridge, Cambridge, United Kingdom

## Abstract

Caenorhabditis elegans is frequently used as a model species for the study of bacterial virulence and innate immunity. In recent years, diverse mechanisms contributing to the nematode's immune response to bacterial infection have been discovered. Yet despite growing interest in the biochemical and molecular basis of nematode-bacterium associations, many questions remain about their ecology. Although recent studies have demonstrated that free-living nematodes could act as vectors of opportunistic pathogens in soil, the extent to which worms may contribute to the persistence and spread of these bacteria has not been quantified. We conducted a series of experiments to test whether colonization of and transmission between C. elegans nematodes could enable two opportunistic pathogens (Salmonella enterica and Pseudomonas aeruginosa) to spread on agar plates occupied by Escherichia coli. We monitored the transmission of S. enterica and P. aeruginosa from single infected nematodes to their progeny and measured bacterial loads both within worms and on the plates. In particular, we analyzed three factors affecting the dynamics of bacteria: (i) initial source of the bacteria, (ii) bacterial species, and (iii) feeding behavior of the host. Results demonstrate that worms increased the spread of bacteria through shedding and transmission. Furthermore, we found that despite P. aeruginosa's relatively high transmission rate among worms, its pathogenic effects reduced the overall number of worms colonized. This study opens new avenues to understand the role of nematodes in the epidemiology and evolution of pathogenic bacteria in the environment.

## INTRODUCTION

Over the last 15 years, the nematode Caenorhabditis elegans has emerged as a model species for the study of pathogen virulence and innate immunity (for examples, see references 1 and 2). Research in this area was originally split between studies of specialist parasites of C. elegans (3) and bacteria relevant to human health: food-borne pathogens such as Salmonella enterica (4) and Listeria monocytogenes (5), opportunistic pathogens such as Pseudomonas aeruginosa (6), and even probiotics (7). Both sides have contributed to an increasingly detailed picture of the nematode's immunity, involving both the nervous system (4) and the intestinal epithelium (8). Although the lack of shared pathways with other animal phyla (2) may hinder the usefulness of C. elegans as a model for biomedical immunology (9), its potential role as a model for environmental health is gaining momentum.

While free-living nematodes have long been recognized as major players in soil ecology (10–12), their ability to carry and shed pathogenic bacteria in the vicinity of crops, livestock, and human populations has been causing some concern for food production (13) and public health (14–16). In particular, C. elegans, a bacterivorous nematode ubiquitous in anthropogenic organically enriched soils (43), provides an exceptionally apt experimental model both in the field and in the laboratory. Thus, it is timely to revisit our understanding of the interactions between C. elegans and food-borne or opportunistic bacterial pathogens from an ecological point of view.

Several factors are expected to contribute to the ecological dynamics of nematode-bacterium associations, combining features from predatory, symbiotic, and parasitic interactions. First, the feeding behavior of nematodes, driven by chemotaxis, governs the opportunity for association. In particular, avoidance of potentially harmful bacteria in the environment has been documented in C. elegans (17) and contributes to the first line of immune defenses for nematodes (18). Second, bacterial strains vary greatly in their ability to survive and grow in the digestive tract of C. elegans (19); and those that successfully colonize the worm's intestine can cause various reductions in the survival of their hosts. The latter feature has been undoubtedly the most extensively studied trait in infection and immunity of C. elegans (1). Whether premature death of infected worms is caused by nutritional deprivation (20) or toxic bacterial products (29), this will affect their ability to spread bacteria over long distances. Third, live bacteria shed by defecation may be ingested by other nematodes, potentially expanding the range of their dispersal in a process akin to fecal-oral transmission of pathogens within populations of larger animals. Years after the proof-of-principle of such bacterial transmission between nematodes has been established (21), very little is known about the factors affecting this process.

Our aim in this study was to start quantifying the fecal-oral transmission of bacterial pathogens between C. elegans nematodes and its contribution to the spread of bacteria in the environment. More specifically, we wanted to investigate how traits from both nematodes and bacteria could affect the success of transmission. When revisiting the ecological framework laid out in the previous paragraph in the context of transmission, we identified three key questions that guided our study design.

First, ingestion of bacteria by C. elegans is driven not only by individual feeding preferences but also by collective behavior (22): in particular, some wild isolates as well as npr-1-defective mutants aggregate into foraging swarms around the edge of bacterial lawns (23) in response to oxygen gradients (24). If another bacterial species was introduced in this environment, we hypothesized that the swarming behavior could have two effects: on the one hand, reduced roaming may decrease the dispersal of bacterial colonies; on the other hand, aggregation may enhance transmission. We tested this by comparing the gregarious strain CB4856 with the canonical nongregarious N2 strain of C. elegans.

Second, opportunities for transmission could be affected not only by the survival of infected nematodes but also by their fecundity, as suggested by experimental fecal-oral transmission of Salmonella enterica from hermaphroditic worms to their offspring (21). Although very few studies have documented variations in the fecundity of C. elegans in response to different bacterial foods (25, 26), we recently found that worms fed on Pseudomonas aeruginosa PAO1 produce around half as many viable offspring as worms fed on either Escherichia coli OP50 or Salmonella enterica Typhimurium JH3010 (S. A. Diaz, E. Mooring, E. G. Rens, and O. Restif, unpublished data). We therefore hypothesized that the transmission success of P. aeruginosa PAO1 in the offspring would be lower than that of S. enterica JH3010.

Third, considering S. enterica and P. aeruginosa as opportunistic pathogens introduced into an environment occupied by E. coli, we asked to what extent colonization of worms and subsequent transmission would actually contribute to the fitness of these opportunistic pathogens. Indeed, under standard laboratory conditions, bacteria replicate both inside and outside worms on agar plates and are therefore limited by competition for space. Hence, we predicted that worms would generally enhance the spread and abundance of the introduced strain of bacteria through gut colonization, shedding, and fecal-oral transmission.

In order to assess the relative importance of these processes, we introduced a third experimental treatment (beside the two pairs of worms and bacterial strains): the same amount of S. enterica or P. aeruginosa bacteria could be introduced either as free-living bacteria on the same plate as a worm fed on E. coli or as an intestinal symbiont inside a worm. In the former case, worms would have to go and graze on that colony before any shedding and transmission could happen. Hence, we hypothesized that the latter treatment would yield both higher transmission success and higher bacterial fitness. The fitness of bacteria was measured by the number of bacteria both inside the worms and on the plates at the end of the experiment and compared to a control with no worms on the plates.

## MATERIALS AND METHODS

### General maintenance and strains.

We used two strains of Caenorhabditis elegans: N2 (Bristol isolate) and CB4856 (Hawaii isolate), obtained from the Caenorhabditis Genetics Centre (CGC) at the University of Minnesota. The two clones were then expanded for approximately 3 generations and subsequently cryopreserved at −80°C in 2.0-ml cryotubes (catalog no. CLS430659; Sigma-Aldrich). Before an experiment, a new tube was thawed and expanded for 1 generation on Escherichia coli (27). In all our experiments, worms were maintained at 25°C and cultured on nematode growth medium (NGM; catalog no. N1000; US Biological). For the bacteria, we used three strains: E. coli OP50-1 (streptomycin-resistant strain), obtained from the CGC; Salmonella enterica serovar Typhimurium JH3010 (chloramphenicol-resistant strain derived from wild-type strain SL1344), provided by Andrew Grant (University of Cambridge); and Pseudomonas aeruginosa PAO1 (a gentamicin-resistant strain), provided by Craig Winstanley (University of Liverpool). Before experiments, bacteria were grown for 16 h in LB broth shaken at 220 rpm at 37°C. LB broth contained the appropriate antibiotic for selection (streptomycin, 50 μg/ml; chloramphenicol, 10 μg/ml; or gentamicin, 100 μg/ml). Unless otherwise mentioned, all agar plates used in the experiments were initially seeded with lawns of E. coli OP50 as the main food source for the nematodes.

### Bacterial transmission assay.

**FIG 1:**
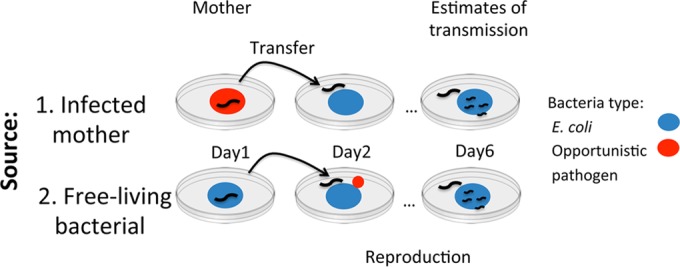
Schematic diagram of the experimental assay, highlighting the two routes of introduction of opportunistic pathogens (S. enterica or P. aeruginosa, in red) onto plates occupied by E. coli (in blue): either inside the intestine of a single worm (1, top) or as a free-living colony alongside a single worm fed on E. coli (2, bottom).

Bacterial transmission was quantified from a mother to its progeny after 6 days of development (Fig. 1). We estimated the variations between the two initial sources (infected mother or environmental source), the two species of opportunistic pathogen (S. enterica and P. aeruginosa), and the two genotypes of the host (C. elegans N2 and CB4856). At the start of the experiment, first-stage larval (L1) age-synchronized worms were generated and arrested by hypochlorite treatment of each worm strain (27). Arrested L1s of each strain were introduced to the standard food (E. coli OP50) or the opportunistic pathogen (Fig. 1). After 24 h of feeding on bacteria, young larvae from both E. coli and pathogen treatments were washed individually with antibiotics (containing both bactericidal and bacteriostatic drugs), using a modified protocol of previously published methods (19). Briefly, larvae were transferred to an unseeded NGM plate containing antibiotics (streptomycin, 50 μg/ml; chloramphenicol, 10 μg/ml; and gentamicin, 100 μg/ml) to remove external bacteria; then, the larvae were paralyzed with a 10-μl drop of filter-sterilized M9 containing tetramisole (500 nM); after 1 min, the larvae were washed twice in a drop of M9 containing the paralyzer and antibiotics; and lastly, we twice washed the larvae in a drop of M9 only in order to remove antibiotics. The bacterial load in the mothers at day 2 was approximately 2.58 ± 0.02 and 3.08 ± 0.08 (mean log_10_ CFU ± standard error of the means [SEM]) for S. enterica- and P. aeruginosa-fed worms, respectively. Individual larvae were then transferred to a petri dish containing the standard food and allowed to develop and produce progeny for 72 h (day 6) (Fig. 1). At this point, the number of progeny was determined as the number of larvae present on the plate per strain and treatment. Mothers were distinguished by their large size and slow movement compared to their progeny. Unless otherwise stated, worms were maintained at 25°C during their development.

### Colonization rate and bacterial load.

The number of colonized worms and their bacterial load were estimated in a random sample of individuals from the progeny at day 6 in parallel with the amount of bacteria present on the plate (Fig. 1). First, the progeny and bacteria present on each plate were collected in a 15-ml Falcon tube. A 3-ml volume of M9 buffer (containing 25 μM tetramisole) and a sterile cell spreader were used for dislodging worms and bacteria. The sample was mixed by vortexing, at which point we collected a subsample of 300 μl of the liquid for estimation of the number of bacteria present in the environment, using serial dilutions (up to 10^−7^). We plated 10^−5^ to 10^−7^ dilutions into selective agar plates (depending on the bacteria) to record the number of bacteria spread into the environment. The remainder of the liquid was mixed with antibiotics and incubated for 1 h with rocking action. After incubation, the sample was centrifuged (1,500 rpm for 1 min) and washed twice with M9 to remove antibiotics. The pelleted sample from each tube with the progeny was then transferred to an agar plate containing antibiotics as before. We then randomly selected 10 worms of similar age (young adults) and washed them two more times with 10 μl of M9 buffer to further remove antibiotics. Individual worms were then picked up and transferred to 2.0-ml Eppendorf tubes (catalog no. 022363352) containing 50 μl of phosphate-buffered saline (PBS). Individual worms were lysed using stainless steel balls and a Mixer Mill MM 300 (catalog no. 85300; Qiagen Retsch TissueLyser) for 1 min at 20 Hz. Individual worm lysates were then added with 450 μl of PBS and diluted in 1:10. Samples from the undiluted and diluted lysates were plated on MacConkey agar to estimate the number of live cells of the pathogenic bacteria and the standard food per worm. Bacterial species were identified according to their lactose-fermenting ability; E. coli is a lactose-fermenting bacterium, while S. enterica and P. aeruginosa are nonfermenting organisms. Both worms and plate samples were incubated overnight at 37°C. After the incubation, we counted the CFU per worm and per plate for each strain and treatment.

### Bacterial growth on plates without worms.

In order to quantify the effects of worms in spreading opportunistic pathogens, we compared bacterial numbers in the bacterial transmission assay to those in a treatment where worms were not present. Plates were seeded with an amount of bacteria similar to the one before on day 2 (Fig. 1) (ca. 2.29 and 3.05 log_10_ CFU per plate for S. enterica and P. aeruginosa, respectively) and incubated until day 6, and the bacteria were harvested using the same protocol.

### Statistical analysis. (i) Proportion of colonized worms and their bacterial load.

Generalized mixed-effect models (GLMM) were used to analyze the variation in the proportion of colonized progeny and their bacterial load in relation to three explanatory variables: (i) the initial source (Source) of the opportunistic pathogen (colonized mother versus free-living source), (ii) the species of bacteria (Bacteria) (S. enterica and P. aeruginosa), and (iii) the worm genotype (Worm) of C. elegans (N2 and CB4856). GLMM with a binomial error distribution (with a logit link function) was used, including a random term for individual worms within plates. For the analysis of the colonization data, the response variable was colonized or not. For the bacterial load, because the load of S. enterica or P. aeruginosa could be affected by the amount of E. coli bacteria present in the intestine of a worm, the response variable was the number of pathogenic CFU in relation to the number of E. coli CFU. For each data set, a series of candidate models were constructed to evaluate the effect of each explanatory variable and their interactions. The models were compared using the Akaike information criterion (AIC). Estimates are reported as means ± SEMs, unless otherwise mentioned. For the analysis of the proportion of worms colonized, the data contained 976 individual observations grouped in 113 plates across variables. For the bacterial load, we included only those worms that were colonized; thus, the data contained 419 individual observations grouped in 76 plates across variables.

### (ii) Analysis of the spread of bacteria on the plate and the mother.

Generalized linear models (GLM) with a binomial error distribution were used to analyze the variation in the amount of opportunistic pathogen and the food source on the plate and the mother. As was done for the bacterial load in the progeny, we included the number of E. coli on the plate and in the mother as response variables. The explanatory variables, model construction, and selection were as described above. Models were fitted separately to the environment and mother data. The data contained 113 plates.

### (iii) Analysis of correlations between bacteria in progeny, mother and plate.

Spearman correlation tests were used to look at the association between the bacterial loads of the opportunistic pathogen in the progeny and in the mother and the spread of the bacteria in the environment. Bonferroni's correction was applied.

## RESULTS

### Nematode fertility.

**FIG 2:**
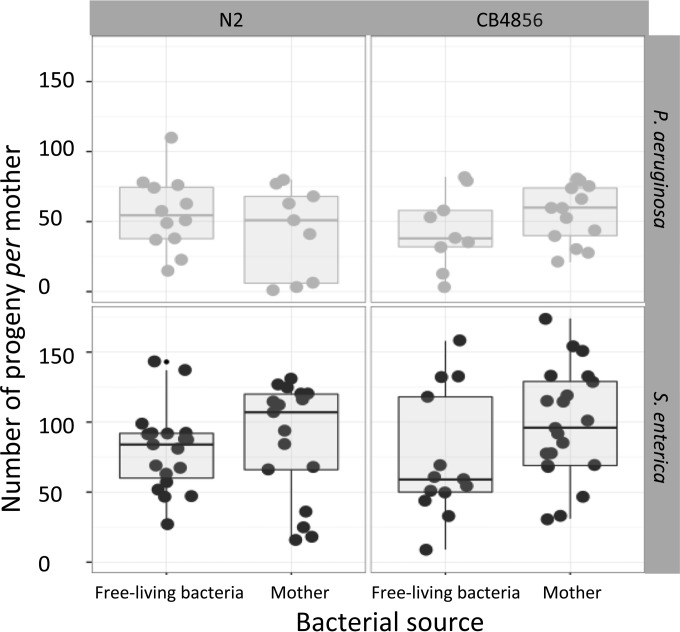
Distribution of the number of progeny produced by each mother in relation to the source of the opportunistic pathogen, the species of bacteria, and the worm genotype. Top and bottom rows show the variation in colonization in worms with P. aeruginosa and S. enterica, respectively. Left and right columns show the variation between C. elegans genotypes (N2 and CB4856).

The best model for the number of larvae produced by mothers only included bacterial species (Tables 1 and 2, model A; Fig. 2): nematodes fed on S. enterica produced on average 40% more offspring than those fed on P. aeruginosa (86.2 ± 4.6 and 51.5 ± 3.9 larvae, respectively).

**TABLE 1:** Model set for response variables^*a*^

Variables in the model	*K*^*b*^ for models B and C	AIC for model:			*K* for models A, D, and E	AIC for model:	
		A	B	C		D	E
Null model	2	1,148	798	14,745	1	15,127	2,526
Source	3	1,148	772	14,726	2	13,894	2,381
Bacteria	3	**1,124**	782	14,742	2	11,685	2,013
Worm	3	1,150	790	14,745	2	9,478	2,377
Source + Bacteria	4	1,124	749	**14,722**	3	10,906	1,712
Source × Bacteria	5	1,124	743	14,724	4	9,876	1,664
Source × Bacteria + Worm	6	1,126	**737**	14,724	5	8,261	1,511
Source × Bacteria + Source × Worm	7	1,126	737	14,725	6	8,196	1,512
Source × Bacteria + Source × Worm + Bacteria × Worm	8	1,128	738	14,727	7	7,862	1,469
Source × Bacteria × Worm	9	1,130	738	14,725	8	**6,530**	**1,440**

a Response variables: A, fertility of the mother; B, number of colonized worms; C, bacterial load in the progeny; D, number of bacteria in plate; E, bacterial load in the mother. The best model for each data set is shown in boldface.

b *K*, number of parameters. For models B and C, plate is included as a random effect (see Materials and Methods).

**TABLE 2:** Summary of the best models listed in Table 1 to describe fertility, the number of colonized worms, and bacterial load in the progeny^*a*^

Model^*b*^	Effects	Estimate	SE or variance^*c*^	t-value, Z-value, or SD	*P* value
A	Fixed				
	Intercept	50.37	5.25	9.58 (t)	<0.001
	Bacteria (S. enterica)	35.85	6.77	5.35 (t)	<0.001
B	Fixed				
	Intercept	−2.41	0.75	−3.23 (Z)	<0.01
	Source (colonized mother)	6.24	1.20	5.21 (Z)	<0.001
	Bacteria (S. enterica)	−1.50	0.92	−1.63 (Z)	0.1
	Worm (CB4856)	1.81	0.66	2.76 (Z)	<0.001
	Source (colonized mother): bacteria (S. enterica)	−3.97	1.45	−2.75 (Z)	<0.001
	Random				
	Plate (intercept)		8.23 (v)	2.87 (SD)	
C	Fixed				
	Intercept	−1.63	0.31	−5.27 (Z)	<0.001
	Source (colonized mother)	1.70	0.33	5.10 (Z)	<0.001
	Bacteria (S. enterica)	−0.81	0.32	−2.51 (Z)	<0.05
	Random				
	Plate (intercept)		1.87 (v)	1.37 (SD)	

a Abbreviations: v, variance; t, t-value; Z, Z-value, SD, standard deviation.

b A, fertility (113 plates); B, no. of colonized worms (967 observations grouped in 113 plates); C, bacterial load in progeny (419 observations in 76 plates).

c All values in this column are SE unless indicated as variance (v).

### Colonization in the progeny. (i) Proportion of colonized worms.

**FIG 3:**
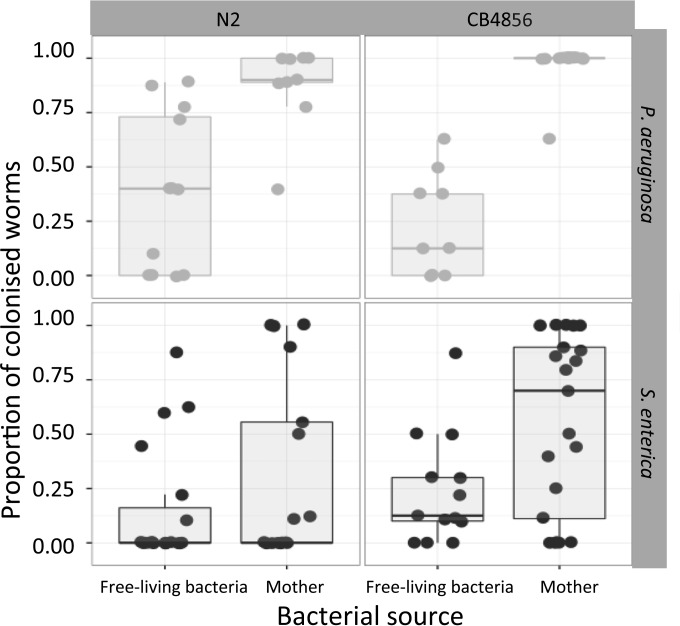
Box plot of the variation in the proportion of colonized progeny by the opportunistic pathogen per plate. Top and bottom rows show worm colonization by P. aeruginosa and S. enterica, respectively. Left and right columns show the variation between C. elegans genotypes (N2 and CB4856). Each dot represents one plate observation.

Of 976 nematode offspring tested across all treatments, 419 were colonized by either opportunistic pathogen by the end of the experiment. The proportion of worms colonized on each plate varied significantly with the source and species of the opportunistic pathogen and the genotype of the worm (best model, Source × Bacteria + Worm) (Tables 1 and 2, model B; Fig. 3). In particular, the progeny of mothers previously grown on an opportunistic pathogen were 2.7 times as likely to be colonized as the progeny on plates where the opportunistic pathogen was initially free-living (averages across data, 0.62 ± 0.05 and 0.23 ± 0.03, respectively) (Fig. 3). P. aeruginosa colonized a higher proportion of the progeny than S. enterica when the mother was previously colonized (averages across data, 0.63 ± 0.06 and 0.32 ± 0.04, respectively). Additionally, there was variation in colonization between C. elegans genotypes: N2 worms showed a lower proportion of colonized progeny than CB4856 worms (averages across data, 0.36 ± 0.05 and 0.52 ± 0.05, respectively).

### (ii) Bacterial load in the progeny.

**FIG 4:**
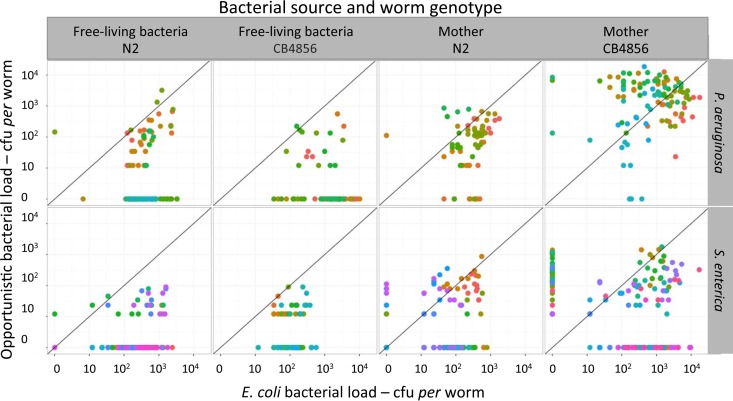
Variation of bacterial load (estimated as CFU) per worm of the progeny, showing the relationship between the observed number of E. coli cells (horizontal axis) and opportunistic pathogens (vertical axis) across treatments. Top and bottom rows show the variation in colonization in worms with P. aeruginosa and S. enterica, respectively. The two left columns show CFU in those worms initially incubated with the free-living bacteria, and the columns on the right show CFU in those worms incubated with a colonized mother. Each dot is an individual worm, color coded by plate.

The average bacterial load per worm ranged from 12 to 7,800 CFU across plates (average, 722 CFU per worm) (Fig. 4). We found that bacterial load varied significantly with the source and the species of bacteria (best model, Source + Bacteria) (Fig. 4; Tables 1 and 2, model C). The average load in progeny of previously colonized mothers was 10 times higher than in progeny on plates with the free-living bacteria (1,110 ± 271 and 104 ± 32 CFU per worm, respectively) (Fig. 4). Additionally, the load in the progeny of infected mothers was 10-fold higher for P. aeruginosa than S. enterica (1,340 ± 336 and 161 ± 43 CFU per worm, respectively).

### Bacteria present on the plate.

**FIG 5:**
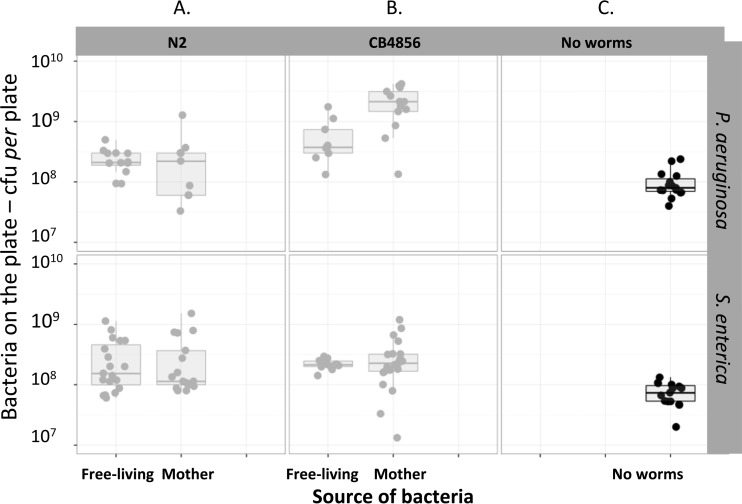
Distribution of the amount of free-living opportunistic pathogens on plates at the end of the experiment. Top and bottom rows show the variation in spread of the opportunistic pathogen into the plate in those worms incubated with P. aeruginosa and S. enterica, respectively. Columns show the variation between plates with C. elegans genotypes N2 (A) and CB4856 (B) and plates without worms (C).

The quantity of opportunistic pathogen on the plates at the end of the experiments varied with the source of the bacteria, the species of bacteria, and the genotype of the worms (best model, Source × Bacteria × Worm interaction) (Fig. 5A and B; Table 1, model D). P. aeruginosa was more abundant than S. enterica on plates where either opportunistic pathogen was introduced by nematodes (14.0 × 10^8^ ± 2.9 × 10^8^ and 3.19 × 10^8^ ± 0.54 × 10^8^ CFU per plate, respectively). S. enterica reached the highest bacterial loads on those plates where the bacteria were initially free-living. The worm genotype affected the amount of opportunistic pathogen on the plate differently; for instance, those plates of CB4856 genotype with the colonized mother showed the highest load on P. aeruginosa compared to all the other treatments (2.17 × 10^9^ ± 3.59 × 10^8^ per plate) (Fig. 5A). Finally, plates with worms had 10 times as many bacteria as plates without worms (best model, Worm presence + Bacteria) (Table 3; Fig. 5C).

**TABLE 3:** Model set for bacterial number in relation to the presence of worms and the bacterial species^*a*^

Variable(s) in the model	*K*^*b*^	AIC
Null model	1	191.64
Worms present	2	160.57
Bacteria	2	184.82
**Worms present + Bacteria**	**3**	**146.43**
Worms present × Bacteria	4	146.60

a The best model is shown in boldface.

b *K*, number of parameters.

### Colonization load in the mother.

**FIG 6:**
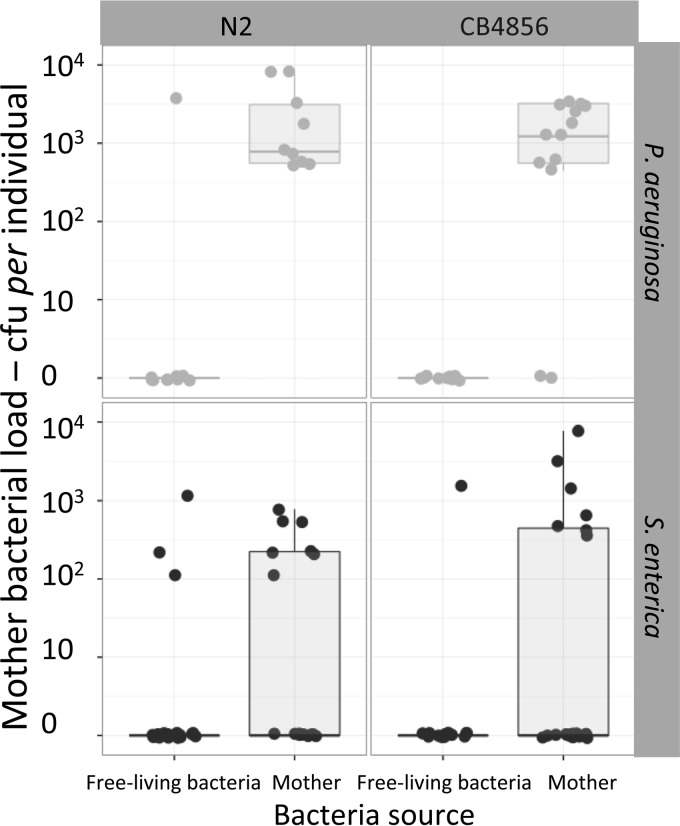
Distribution of pathogen load per mother in relation to variables source, bacteria, and worm. Top and bottom rows show CFU in mothers incubated with P. aeruginosa and S. enterica, respectively. Left and right columns show the variation between C. elegans genotypes (N2 and CB4856). Each dot represents observations for each mother per plate.

There was a large variation in the colonization among mothers by the opportunistic pathogen, ranging between 0 and 77.70 × 10^2^ CFU per mother. The source and species of bacteria and worm genotype affected the bacterial load in the mother (for Source × Bacteria × Worm, AIC = 1,440) (Fig. 6; Table 1, model E). The average load of S. enterica was 4.5 times higher in mothers initially fed on the opportunistic pathogen than in mothers initially fed on E. coli (447 ± 220 and 97.22 ± 61.60 CFU per mother, respectively); the ratio was 17 for P. aeruginosa (2,060 ± 220 and 180 ± 179 CFU per mother, respectively). Regarding worm strains, N2-colonized mothers showed a higher average bacterial load than did CB4856-colonized mothers and in response to P. aeruginosa than to S. enterica (2.61 × 10^3^ ± 1.01 × 10^2^ CFU per mother) (Fig. 6).

### Correlations between bacterial loads in progeny and in mother and bacteria present on the plate.

Finally, we assessed whether the bacterial load in the progeny was associated with the bacterial load present in the mother or in the plate. After correcting for multiple testing, we did not find a significant correlation between bacterial load in the progeny, bacterial load in the mother, and bacteria present on the plate between worm strains, bacterial species, or bacterial source (see Table S1 in the supplemental material). We found that the plates with CB4856 genotype harboring the highest loads of P. aeruginosa in the progeny also had the highest bacterial loads in the environment, suggesting a positive feedback between the two factors; however, this relationship was not significant or ubiquitous.

## DISCUSSION

In this study, we investigated the effects of C. elegans nematodes on the dynamics of pathogenic bacteria S. enterica and P. aeruginosa. Our results demonstrate that the presence of worms contributes to the spread of these opportunistic pathogens under experimental conditions in which mainly E. coli is present as a food source. Specifically, the total amount of opportunistic pathogens in the environment was on average 10 times as high on plates containing worms than on plates with bacteria only. Our results also demonstrate that transmission between worms is an efficient source of enteric colonization by bacteria. The number of colonized worms was twice higher and the bacterial load 10 times higher when the initial bacterial source was an infected mother than with free-living bacterial treatment.

We have provided the first joint quantification of within- and between-worm bacterial dynamics, demonstrating that transmission from mother to offspring yields greater numbers of bacteria. Using fluorescence microscopy, Kenney et al. (21) observed that mothers infected with S. enterica could infect their progeny by three routes: within the intestine when eggs hatched internally, by direct contact when progeny fed on bacteria bursting out of dead mothers, or indirectly by fecal-oral environmental transmission. All the mothers included in our analysis survived to the end of the experiment, which allows us to dismiss the first two routes of transmission. In addition to fecal-oral transmission between live worms, bacteria can become attached to a worm's cuticle (28) and be either released later or ingested by another worm. However, the latter route of transmission is unlikely to have contributed to the initial seeding of P. aeruginosa or S. enterica when infected mothers were transferred to E. coli plates, as we washed them in antibiotic solutions that would have killed most bacteria attached to the cuticle.

By comparing two worm genotypes and two bacterial species, we were able to test three questions about the relative effects of these variables on the dynamics of bacteria in nematode populations. First, we hypothesized that the feeding behavior of worms would affect the dispersal and transmission of bacteria. Indeed, bacterial colonization of worms in our experiments was 40% higher among the gregarious CB4856 isolate of C. elegans than among standard N2 worms. However, colonized worms from either genotype carried similar bacterial loads. This suggests that the swarming behavior of CB4856 increased the exposure of worms to bacteria but had no effect on the within-host dynamics.

Second, we expected that P. aeruginosa would achieve lower transmission success from mother to offspring than S. enterica as a consequence of the former reducing worm fecundity. Indeed, we found that those mothers initially fed with P. aeruginosa produced 40% less progeny than those fed on S. enterica. Further, the same effect was observed among mothers initially fed on E. coli and transferred to plates containing a patch of P. aeruginosa, even though most of them did not get colonized (Fig. 6). Thus, the reduction in fecundity may have been caused by the presence of toxic bacterial products (29). Taking into account both worm fecundity and bacterial transmission from mother to its offspring, the absolute number of infected worms was higher for S. enterica than P. aeruginosa (average colonization rates, 27 of 86 worms and 16 of 32 worms, respectively).

Our third objective was to compare the dispersal of S. enterica and P. aeruginosa by worms in an environment where E. coli is an abundant food source. We found that P. aeruginosa grew to levels four times as high as S. enterica on those plates where the opportunistic pathogen was introduced by worms. The spread of these pathogens did not affect E. coli abundance (see Fig. S1 in the supplemental material). Further, we also found an interaction between worm genotype and bacterial species, with P. aeruginosa achieving the highest growth in the presence of CB4856 worms.

### Bacterial dynamics.

P. aeruginosa and S. enterica exhibited strong differences in their abilities to colonize mothers and their progeny, which could reflect variations in both within-host and between-host dynamics. In line with previous studies (19, 30, 31), we found that adult worms could harbor 10^2^ to 10^4^ live bacteria in their intestine. Although several studies have reported genetic and environmental factors affecting bacterial loads in C. elegans, the relative contributions of ingestion, enteric growth, and shedding of bacterial cells to variations in bacterial numbers remain largely unknown. At the uptake stage, it has been previously shown that some bacterial cells may escape mechanical degradation by the pharyngeal grinder (32), with small cells potentially escaping more easily (33). Then, there is differential colonization and proliferation in the intestine of worms depending of the bacterial species (19, 30). A recent study monitoring worms from the age of 2 to 6 days reported increases in enteric bacterial load from 10^2^ to 10^4^ with E. coli, whereas worms feeding on S. enterica eventually reached a bacterial load of 10^5^ (19). Although the latter species did not grow to such high levels in our study (possibly due to strain-specific differences), we observed a 10-fold increase in P. aeruginosa over S. enterica (Fig. 4).

Another factor potentially affecting bacterial dynamics is competition between strains or species. Portal-Celhay et al. (30) showed that even a single short exposure to S. enterica followed by transfer to E. coli was sufficient to maintain a 10% bacterial load by the former after 48 h. In our study, we found that the infected progeny contained an average bacterial load of 40% S. enterica and 50% P. aeruginosa. These greater levels of colonization load suggest a potential contribution of reinfection involving fecal-oral transmission. In a different setting, such cycles of reinfection have been shown to play an important role in maintaining enteric colonization by Campylobacter jejuni in farmed chickens (34). In our study, the two bacterial species grew on the plates at similar rates in the absence of worms (averages after 5 days of incubation, 7.95 ± 0.05 and 7.83 ± 0.05 log_10_ CFU for P. aeruginosa and S. enterica, respectively) (Fig. 5C). Therefore, the higher bacterial numbers produced in the presence of worms provide clear evidence that colonization and transmission contribute to the fitness of these opportunistic pathogens in this system.

### Differences between worm genotypes.

We found differences in transmission between the two genotypes of C. elegans. Progeny of CB4856 were nearly twice as likely to be colonized as the canonical N2 strain. Behavioral differences between the N2 and CB4856 wild-type worms are potentially driving the observed differences. The low oxygen concentration around the edge of bacterial lawns attracts worms of CB4856 and many other wild-type isolates (23), resulting in a seemingly gregarious behavior, whereas N2 worms show an even distribution across the lawn (35). Previous studies indicate that N2 worms are equally attracted to P. aeruginosa PAO1 and E. coli (36), but we cannot rule out a higher attraction toward P. aeruginosa in CB4856 worms. However, if the observed growth rates of P. aeruginosa and S. enterica (Fig. 5C) amount to similar oxygen consumption by the two bacteria, other chemical cues would have to be invoked. Another possible explanation is that CB4856 worms may initially aggregate in clumps, later attracting other potentially already colonized individuals. In both scenarios, the behavior is likely to create spots of high risk for transmission that are absent in N2 worm populations under the tested conditions. An important caveat is that other genetic differences between CB4856 and N2 might play a role in the phenotypes reported here. Further experiments with specific mutant lines will be necessary to establish the genetic determinants of the different aspects of nematode-bacterium interactions.

### Ecology of C. elegans and other free-living nematodes.

Recent research has begun to unveil the natural ecology of free-living nematodes (and in particular C. elegans) and their associations with soil bacteria and other microorganisms (37–40). Experimental studies in soil confinement suggest that C. elegans can harbor a very diverse group of microbes compared to other free-living nematodes such as Acrobeloides maximus (37). Although C. elegans has not been directly associated with food-borne pathogens in natural environments, it has been shown experimentally to be capable of transporting S. enterica from manure to vegetables (15) and also to have the potential to carry food-borne pathogens into vertebrates (41, 42). Our findings further demonstrate that, more than a mere mechanical vector bacterium, C. elegans has the potential to favor the environmental spread of some pathogenic bacteria over others, depending on their relative abilities not only to colonize individual worms but also to transmit within populations.

In summary, we have quantified bacterial transmission between worms and worm contribution to bacterial fitness. We found that the more-pathogenic bacteria exhibited higher transmission rates but also reduced worm reproduction. We also found that a gregarious worm genotype enhanced bacterial transmission. These differences could have consequences in the persistence and evolution of pathogenic bacteria, which merit further study to understand the role of nematodes in the health of ecosystems. A next step will be to validate these results in soil mesocosm, where limiting resources for both bacteria and worms as well as spatial heterogeneity could result in different dynamics and selective pressures at different scales.

## Supplementary Material

Supplemental material
