# Supplementary material for: Spread and Transmission of Bacterial Pathogens in Experimental Populations of the Nematode Caenorhabditis elegans
Source: Appl Environ Microbiol. 2014 Sep;80(17):5411–8. doi: 10.1128/AEM.01037-14 (PMC4136108; doi:10.1128/AEM.01037-14)
Supplement: Supplemental material [file supp_80_17_5411__index.html]

Spread and Transmission of Bacterial Pathogens in Experimental Populations of the Nematode Caenorhabditis elegans — Supplemental material 

# Spread and Transmission of Bacterial Pathogens in Experimental Populations of the Nematode Caenorhabditis elegans

## Supplemental material

**Files in this Data Supplement:**

- Supplemental file 1 -

  Associations between bacterial load in the progeny and bacterial load present on the plate and in the mother for each group (Table S1); distribution of the amount of *E. coli* on plates at the end of the experiment (Fig. S1).

  PDF, 700K
