# Supplementary material for: Spread and Transmission of Bacterial Pathogens in Experimental Populations of the Nematode Caenorhabditis elegans
Source: Appl Environ Microbiol. 2014 Sep;80(17):5411–8. doi: 10.1128/AEM.01037-14 (PMC4136108; doi:10.1128/AEM.01037-14)

Supplementary Information (SI)

Table S1. Associations between bacteria load in the progeny and the bacteria load present on the plate and mother for each group (*Bacteria*: *P. aeruginosa* and *S. enterica*, *Source*: Free bacteria and colonised mother and *Worm*: N2 and CB4856). Speraman rank correlation coefficient (rho) and p-value show significant correlations (shaded cells), otherwise p-values is only shown. * shows significant correlations after Bonferroni correction. NA indicates there were no worm colonisations in one of the groups.

	P. aeruginosa				*S. enterica*			
	Free bacteria		Colonised mother		Free bacteria		Colonised mother	
	Plate	Mother	Plate	Mother	Plate	Mother	Plate	Mother
N2	p=0.55	p=0.82	p=0.58	rho=0.68; p=0.04	p=0.80	p=0.87	rho=0.83; p=0.01	p=0.13
CB4856	p=0.47	NA	rho=0.66; p=0.01	p=0.20	p=0.77	p=0.75	p=0.51	p=0.48

Figure S1. Distribution of the amount of *E. coli* on plates at the end of the experiment. Top and bottom row show the variation in spread of the opportunistic pathogen into the plate in those worms set up with *P. aeruginosa* and *S. enterica*, respectively. Panels show the variation between *C. elegans* genotypes A) N2 and B) CB4856 and C) without worms.

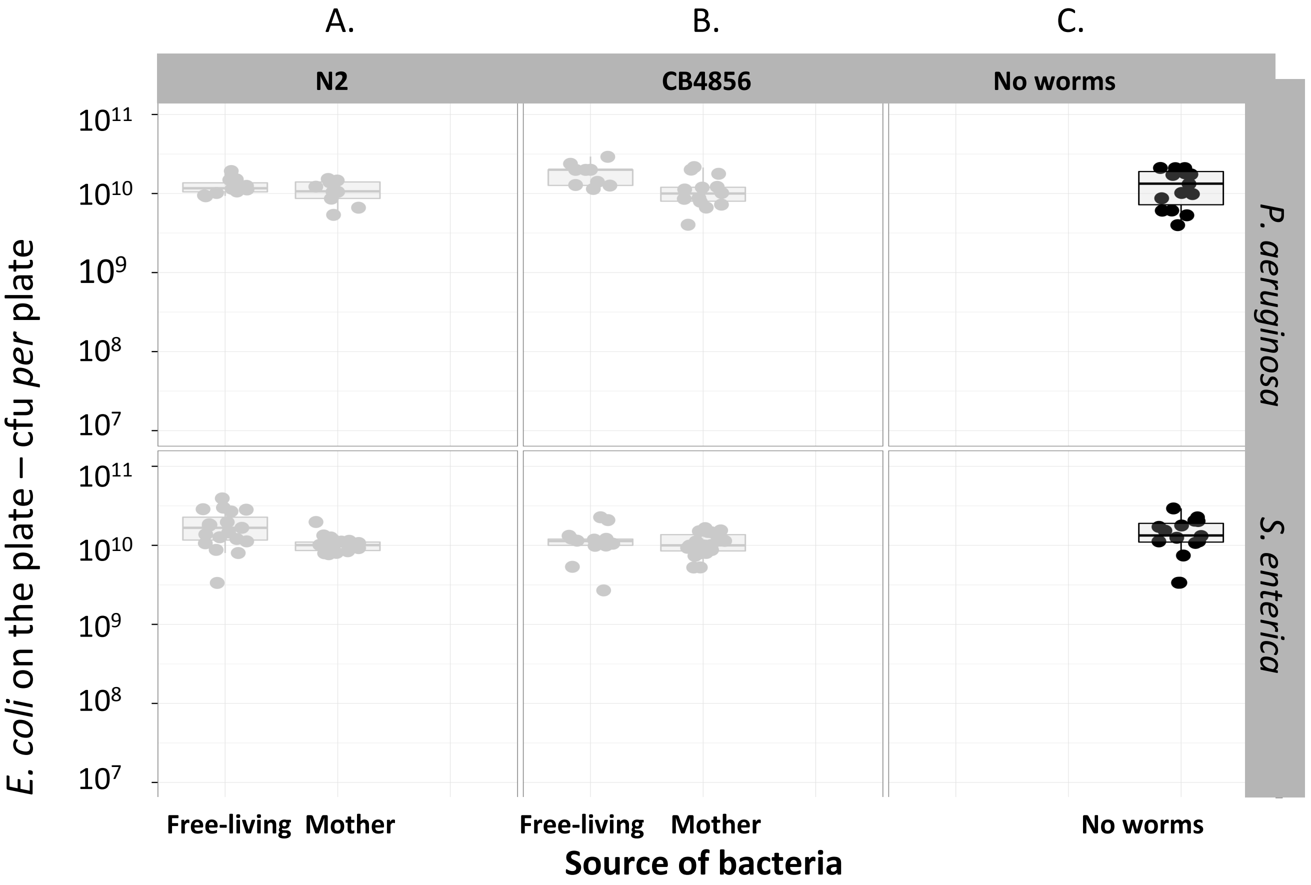

Supplement: Supplemental material [file AEM.01037-14_zam999105616so1.pdf]
